# Systemic Immune-Inflammatory Index, Tumor-Infiltrating Lymphocytes, and Clinical Outcomes in Esophageal Squamous Cell Carcinoma Receiving Concurrent Chemoradiotherapy

**DOI:** 10.1155/2023/4275998

**Published:** 2023-05-16

**Authors:** Jun Yang, Jifang Zheng, Jianjian Qiu, Mengyan Zhang, Lingyun Liu, Zhiping Wang, Qunhao Zheng, Yanyan Liu, Mingqiu Chen, Jiancheng Li

**Affiliations:** ^1^Department of Radiation Oncology, Clinical Oncology School of Fujian Medical University, Fujian Cancer Hospital, No. 420 Fuma Road Jin'an District, Fuzhou 350014, Fujian Province, China; ^2^Clinical Oncology School of Fujian Medical University, No. 420 Fuma Road Jin'an District, Fuzhou 350014, Fujian Province, China

## Abstract

**Background:**

Systemic inflammation may be involved in the entire cancer process as a promoter and is associated with antitumor immunity. The systemic immune-inflammation index (SII) has been shown to be a promising prognostic factor. However, the relationship between SII and tumor-infiltrating lymphocytes (TIL) have not been established in esophageal cancer (EC) patients receiving concurrent chemoradiotherapy (CCRT).

**Methods:**

Retrospective analysis of 160 patients with EC was performed, peripheral blood cell counts were collected, and TIL concentration was assessed in H&E-stained sections. Correlations of SII and clinical outcomes with TIL were analyzed. Cox proportional hazard model and Kaplan–Meier method were used to perform survival outcomes.

**Results:**

Compared with high SII, low SII had longer overall survival (OS) (*P* = 0.036, hazard ratio (HR) = 0.59) and progression-free survival (PFS) (*P* = 0.041, HR = 0.60). Low TIL showed worse OS (*P* < 0.001, HR = 2.42) and PFS (*P* < 0.001, HR = 3.05). In addition, research have shown that the distribution of SII, platelet-to-lymphocyte ratio, and neutrophil-to-lymphocyte ratio were negatively associated with the TIL state, while lymphocyte-to-monocyte ratio presented a positive correlation. Combination analysis observed that SII^low^ + TIL^high^ had the best prognosis of all combinations, with a median OS and PFS of 36 and 22 months, respectively. The worst prognosis was identified as SII^high^ + TIL^low^, with a median OS and PFS of only 8 and 4 months.

**Conclusion:**

SII and TIL as independent predictors of clinical outcomes in EC receiving CCRT. Furthermore, the predictive power of the two combinations is much higher than a single variable.

## 1. Introduction

Among the 10 leading causes of cancer death in the world, esophageal cancer (EC) is the sixth and accounts for 5.56% of cancer-related deaths [1]. The incidence of EC varies considerably by region and ethnicity, and this also determines the prevalence of potential risk factors and the distribution of subtypes [2]. Because initial symptoms are often ignored, most patients are diagnosed with progressive disease, negating the possibility of surgical intervention. However, the 5-year overall survival (OS) rate for EC is ∼16% [3]. In countries with high incomes, it is much higher from 24% to 36% [2]. This mainly belonged to the variety of treatments, especially the introduction of immune checkpoint inhibitors (ICIs) [4], which create new opportunities for the treatment of EC.

Response rates to ICIs have been correlated with programed cell death-ligand one expression, and this has become commonplace in clinical practice. In addition, based on a study in malignant melanoma, it was found that tumor-infiltrating lymphocytes (TIL) can be used as a reliable marker to predict the effective rate of ICIs treatment [5]. Subsequent studies also found that TIL-positive cases had significantly better OS than TIL-negative cases when compared with melanoma, breast, lung, liver, and colorectal cancers [6, 7]. Correlation of TIL with prognosis has been demonstrated in EC in recent years. In a study that collected 305 EC, TIL-positive cases were not only associated with CD8^+^ expression but showed a longer OS compared with negative cases [8]. Although this finding was also confirmed by a meta-analysis, CD3^+^, CD4^+^, and CD45RO^+^ TIL were not associated with EC prognosis [9]. Given the diversity of TIL participants, complexity of function, and high cost of research, its role in cancer immunity is unfortunately often underestimated.

Inflammation can be involved in all stages of cancer, from the onset of early gene mutations to tumor development and distant metastasis [10, 11]. And it has been recognized as a potential mechanism of immune resistance against tumors [12]. Recently, markers of inflammation can be characterized by readily available peripheral blood parameters, including neutrophils, lymphocytes, monocytes, and platelets. One of the most commonly used parameters is the inflammatory index, which includes a set of subsets of systemic immune-inflammation index (SII), platelet-to-lymphocyte ratio (PLR), neutrophil-to-lymphocyte ratio (NLR), and lymphocyte-to-monocyte ratio (LMR). Valuable previous studies have shown that SII, calculated as the number of neutrophils, lymphocytes, and platelets, reflects a balance between the host's inflammatory and immune status, noticing as an important prognostic factor in the lung [13], breast [14], gastric [15], esophageal [16], colon [17], and bladder cancers [18]. In patients with resectable adenocarcinoma of the gastroesophageal junction, SII can be considered an independent poor prognostic factor with or without neoadjuvant therapy, a higher level of SII is associated with a lower OS and disease-free survival (DFS) [19]. SII has also been shown to be an easily accessible and useful prognostic marker in patients with surgically resected esophageal squamous cell carcinoma (ESCC). It had predictive power for OS and DFS in I–II stage subgroup, but not for OS in III stage subgroup [20]. In the context of those studies, further confirmation of the predictive power of SII in EC populations receiving definitive chemoradiotherapy is needed. In fact, current research is mostly focused on the local response of lymphocytes in the tumor tissue or the systemic inflammatory response. Theoretically, systemic inflammatory responses could influence the immune microenvironment of tumors to modulate biological processes. Therefore, this study aimed to investigate the relationship between SII and TIL, proving whether the two parts have synergistic effects in predicting prognosis.

## 2. Methods

### 2.1. Patients and Therapy

From February 2014 to December 2021, the clinical data and outcomes of 160 patients with EC who received concurrent chemoradiotherapy (CCRT) were collected in a retrospective database of Fujian Cancer Hospital. All patients had a pathologic diagnosis of EC. Tumor staging was determined according to the American Joint Committee on Cancer (AJCC) 7th edition staging system. Inclusion criteria for this study: (a) pathologically confirmed ESCC in tissue samples; (b) Eastern Cooperative Oncology Group (ECOG) score of 2 or lower; (c) no significant distant metastases were detected; (d) no other tumors. Exclusion criteria: (a) missing data; (b) severe heart, liver, and kidney disease; (c) autoimmune diseases; (d) chronic infectious diseases; (e) receiving corticosteroids or immunosuppressant therapy; (f) hematological disease. Patients who received CCRT were treated according to the Fujian Cancer Hospital standard after diagnosis. Platinum-based combinations with paclitaxel/docetaxel or cisplatin plus 5-fluorouracil were used for chemotherapy. Radiation therapy was based on 3D conventional radiation therapy and intensity-modulated radiation therapy techniques and delivered using six megavolt X-rays. The daily fractions of treatment were 1.8–2.0 Gray (Gy), with total doses ranging from 40 to 66 Gy over 5 or 7 weeks. The period from diagnosis to death of the observed individual was defined as OS. Progression-free survival (PFS) was defined as the period from diagnosis to disease progression. Experiments involving humans were carried out in accordance with the ethics policy approved by the Ethics Committee of Fujian Cancer Hospital (No. YKT2021-005-01). Written informed consent was obtained from all patients.

### 2.2. TIL Assessment

Pretreatment hematoxylin and eosin sections were performed by two experienced pathologists blinded to the clinicopathological information, using the relevant recommendations from the International Immuno-Oncology Biomarkers Working Group for the evaluation of TIL [21]. Tumor-infiltrating lymphocytes (TIL) were centrally assessed by viewing pretreatment H&E sections using a standard light microscope under a field of view at magnification ×200. The percentage of TIL was recorded for each analysis area. A total of 3–5 valid fields of view per slice were randomly selected and the average infiltrating rate was used as the actual TIL score.

### 2.3. Evaluation and Definition

One week before the initiation of the first treatment, the patient underwent peripheral bleeding, routine blood count, and biochemical evaluation. Observed indicators included, but were not limited to, platelets, lymphocytes, neutrophilS, and monocytes. And from these variables, the inflammatory index was calculated. Platelet-to-lymphocyte ratio (PLR) was defined as the ratio of platelet count to lymphocyte count; the count of neutrophils divided by the count of lymphocytes was the neutrophil-to-lymphocyte ratio (NLR); the lymphocyte-to-monocyte ratio (LMR) was calculated as the ratio of the count of lymphocytes to the count of monocytes; immune-inflammation index (SII) was derived based on NLR values multiply by platelet count. Optimal cutoff was calculated using X-tile version 3.6.1 (Yale University, Connecticut, USA) [22]. Hematology indicators were sent to the research department for professional verification according to hospital standards.

### 2.4. Statistical Analyses

The *χ*^2^ and Fisher's exact test were employed to compare differences in categorical variables. For continuous variables, it was expressed as mean ± standard deviation. Differences were compared by one-way analysis of variance. Survival curves were presented using the Kaplan–Meier model. Calibration curve evaluation was used to verify the prognostic predictive ability of the SII. Patients were analyzed using multivariate Cox regression to assess the role of different variables on OS and PFS. Hazard ratio (HR) and 95% confidence intervals (CI) were calculated together. The study also defined the *P* threshold, which means that two-sided *p* < 0.05 was considered statistically significant. All statistical analysis in this research was performed using R ver. 3.6.3 (Vienna, Austria) and SPSS ver. 22.0 (SPSS Inc., Chicago, IL).

## 3. Results

### 3.1. The Relationship of SII and Clinicopathological Characteristics

The clinicopathologic characteristics of 160 patients with EC are shown in Table 1. There were 38 (23.8%) female patients and 122 (76.2%) male patients. In this study, 111 (69.4%) and 49 (30.6%) patients were younger than 70 years and older than 70 years, respectively. In the tumor, nodes, metastasis (TNM) staging assessment, the proportion of patients with T4 was 45.6% and 37.5% of patients with one or two regional lymph node metastases. At the time of diagnosis, the proportion of patients with distant metastases was 19.4%. The proportions of patients ≥15% and <15% in the TIL analysis were 50% each. The optimal threshold values for SII, PLR, NLR, and MRL were 1036.6, 100, 2.32, and 2.5, respectively. SII was significantly associated with tumor length (*P* = 0.016), PLR (*P* = 0.005), NLR (*P* < 0.001), and LMR (*P* < 0.001). However, there were no significant differences according to gender, age, Eastern Cooperative Oncology Group score, TNM stage, and chemotherapy administration.

### 3.2. SII and Survival Outcomes

**Figure 1:**
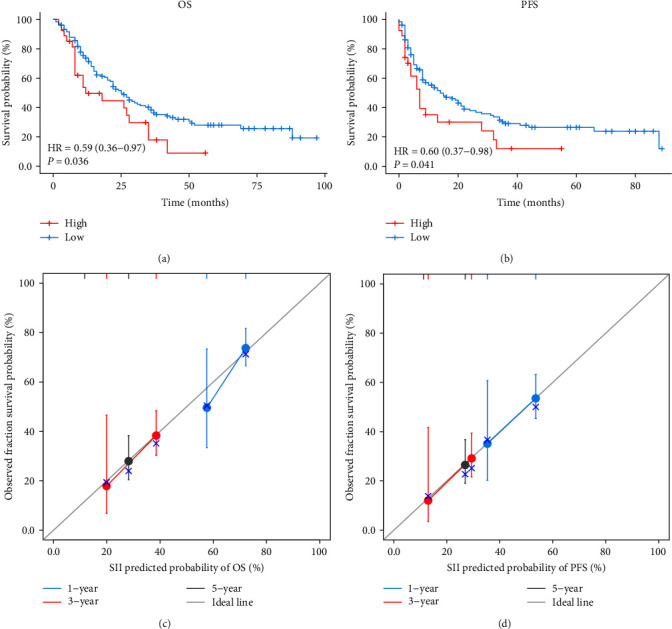
Survival analysis in patients with esophageal cancer receiving concurrent chemoradiotherapy. (a) Kaplan–Meier survival curve for OS according SII. (b) Kaplan–Meier survival curve for PFS according SII. (c) 1-, 3-, and 5-year calibration curves of SII in OS. (d) 1-, 3-, and 5-year calibration curves of SII in PFS. OS, overall survival; PFS, progression-free survival; SII, systemic immune-inflammation index; HR, hazard ratio.

We first divided SII into two groups, high and low, according to the optimal cut-off results, and investigated the prognostic values for OS and PFS in these two groups. Compared with the high group, the results showed that OS of the low group was significantly prolonged. Median survival time was 12 months (range from 8 to 35 months) in the high group and 25 months (range from 21 to 36 months) in the low group (Figure 1(a)). In addition, the 1-, 2-, and 3-year OS rates in the high group were 49.6%, 44.6%, and 17.8%, respectively. The 1-, 3-, and 5-year OS rates in the low group were 73.6%, 38.3%, and 28.0%. PFS analysis showed similar results to OS. We calculated a median survival of 7 months (range from 4 to 32 months) in the high group and a relatively longer median survival of 14 months (range from 9 to 22 months) in the low group (Figure 1(b)). Besides, PFS rates at 1 and 2 years in the high group were 35.6% and 30.1%. In the low group, the 1-, 3-, and 5-year PFS rates were 53.5%, 29.1%, and 26.5%, respectively (Figure 1(b)). Then, the calibration curve results at 1-, 3-, and 5 year showed good predictive ability of OS and PFS in SII (Figures 1(c) and 1(d)).

### 3.3. Multivariate Survival Analysis for Various Variables

**Figure 2:**
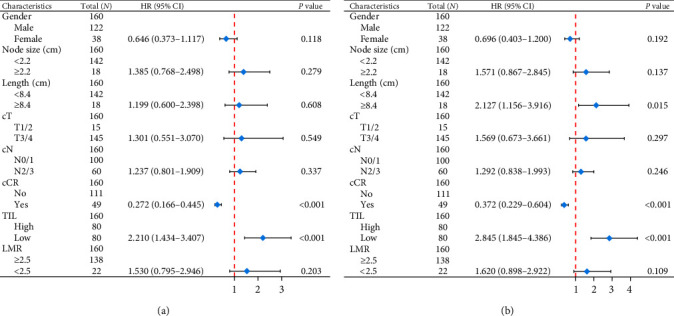
Multivariate survival analysis of OS (a) and PFS (b). OS, overall survival; PFS, progression-free survival; T, tumor; N, node; M, metastasis; cCR, clinical complete response; TIL, tumor-infiltrating lymphocytes; LMR, lymphocyte-to-monocyte ratio; HR, hazard ratio; CI, confidence interval.

In this study, multivariable stratified analysis was performed, and data were derived from the multivariate survival analysis. We conducted a multivariate analysis of OS in patients with EC. A better prognosis was suggested in 49 (30.6%) patients who achieved clinical complete response (cCR) (HR: 0.272; CI: 0.166–0.445; *P* < 0.001). This fully corresponds to the state of clinical practice. In TIL stratified analysis, patients with low infiltration rates (TIL < 15%) were associated with higher mortality (HR: 2.210; CI: 1.434–3.407; *P* < 0.001). Statistically significant values were not observed in other variables, including gender, lymph node size, tumor stage (T stage), nodes stage (N stage), and LMR (Figure 2(a)). In addition, we also performed multivariate survival analysis for PFS (Figure 2(b)). The study showed that tumor length ≥ 8.4 cm (HR: 2.127; CI: 1.156–3.916; *P* = 0.015) and low TIL level (HR: 2.845; CI: 1.845–4.386; *P* < 0.001) were significantly associated with worse PFS. Besides, the presence of cCR (HR: 0.372; CI: 0.229–0.604; *P* < 0.001) was associated with a better prognosis, consistent with OS results.

### 3.4. Distribution of TIL in the EC Population

**Figure 3:**
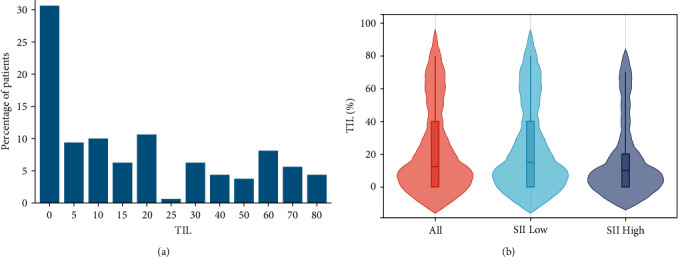
Distribution of TIL in esophageal cancer and various SII populations. (a) The percentage of patients with different concentrations of TIL. (b) The distribution of TIL in the whole, low, and high SII populations. SII, systemic immune-inflammation index; TIL, tumor-infiltrating lymphocytes.

Histograms were used for visualization to clearly define the distribution of TIL in the entire population. It is worth noting that TIL 0% was the highest among all groups, ∼30.63% (49 cases), while the group with TIL 25% had the lowest rate of 0.63% (one case). The highest percentage of TIL was 80% and included seven cases (4.38%) (Figure 3(a)). In addition, the proportion of TIL in different populations was also observed and presented as mean ± standard deviation. In the whole population, low and high SII populations were 22.50% ± 25.27%, 23.76% ± 25.73%, and 16.30% ± 22.30%, respectively. The results showed that the proportion of TIL was lower in the high SII population. However, statistical analysis showed no significant difference (*P* > 0.05) (Figure 3(b)).

### 3.5. Prognostic Significance of TIL

**Figure 4:**
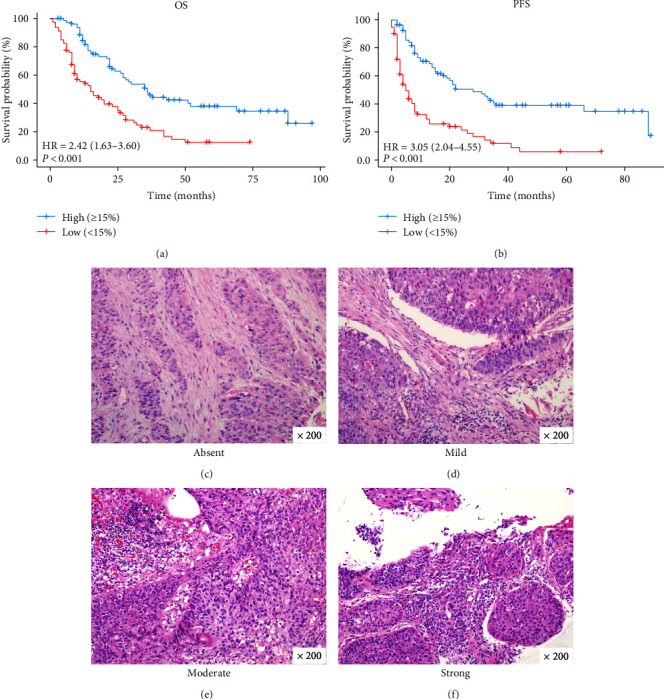
TIL correlated with clinical outcomes. Kaplan–Meier curves for OS (a) and PFS (b) according to TIL. TIL status of absent (c), mild (d), moderate (e), and strong (f). Original magnification, ×200. OS, overall survival; PFS, progression-free survival; HR, hazard ratio.

To demonstrate the correlation between TIL and clinical outcome, Kaplan–Meier analysis showed that OS was significantly longer in high TIL than in the low group (*P* < 0.001). Median survival was 36 months (range, 27–69 months) in the high (≥15%) group and 15 months (range, 10–25 months) in the low (<15%) group. The 1-, 3-, and 5-year OS rates in the high group were 84.3%, 47.4%, and 38.0%, while the 1-, 2-, and 3-year OS rates in the low group were 55.4%, 37.9% and 22.9% (Figure 4(a)). Low TIL was also found to be associated with worse PFS in subsequent analysis (*P* < 0.001). Median survival time was 5 months in the low group and 28 months in the high group. The 1-, 2-, and 3-year PFS rates in the low group were 29.2%, 21.4%, and 11.9%, and the 1-, 3-, and 5-year PFS rates in the high group were 70.3%, 39.0%, and 34.7% (Figure 4(b)). In addition, TIL status was classified into absent, mild, moderate, and strong according to the TIL ratio and visualized by H&E staining sections (Figure 4(c)–4(f)).

### 3.6. Correlation of Different Status of TIL with Inflammatory Index

**Figure 5:**
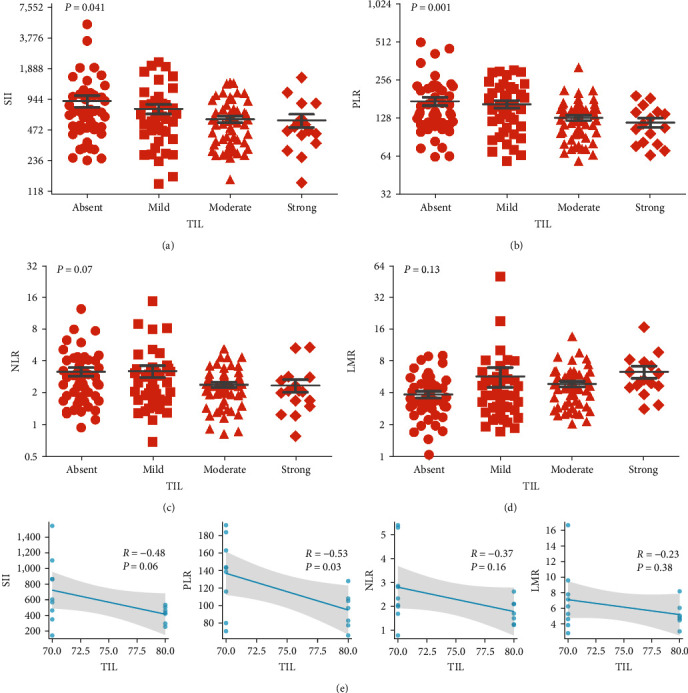
Correlation of different status of TIL with inflammatory index. (a) Relationship between TIL status and SII. (b) Relationship between TIL status and PLR. (c) Relationship between TIL status and NLR. (d) Relationship between TIL status and LMR. *P* values were determined by one-way analysis of variance. (e) In the TIL strong group, Spearman's correlation analysis was used to show the correlation between SII, PLR, NRR, LMR, and TIL. TIL, tumor-infiltrating lymphocytes; SII, systemic immune-inflammation index; PLR, platelet-to-lymphocyte ratio; NLR, neutrophil-to-lymphocyte ratio; LMR, lymphocyte-to-monocyte ratio.

Given the inflammatory index was derived from immune-related cells in the peripheral blood, the inflammatory index may influence the composition of the immune microenvironment of the primary tumor. SII was significantly negatively correlated with TIL status, and the results showed that SII (*P* = 0.04) in tumor tissues with strong TIL was significantly lower than in patients with absent, mild, or moderate tumors (Figure 5(a)). And this phenomenon was further confirmed in PLR (*P* = 0.001) (Figure 5(b)) and NLR analysis (*P* = 0.07) (Figure 5(c)). Conversely, LMR was significantly positively correlated with TIL status, and LMR was significantly higher in TIL-strong tumor tissue than in absent (*P* = 0.02) and moderate tumors (*P* = 0.01) (Figure 5(d)). Although the LMR score for mild TIL showed a higher trend than absent, they were not statistically significant. In addition, we used Spearman's correlation analysis in the TIL strong group to show that SII, PLR, NLR, and LMR were negatively correlated with TIL. There were significant differences between PLR and TIL strong group (*R* = −0.53, *P* = 0.03), while SII and TIL strong group were not significant (*R* = −0.48, *P* = 0.06) (Figure 5(e)). All these data suggested that TIL status was influenced to some extent by inflammatory markers and played a relevant role in remodeling the immune microenvironment.

### 3.7. Association of TIL with Prognosis According to SII

**Figure 6:**
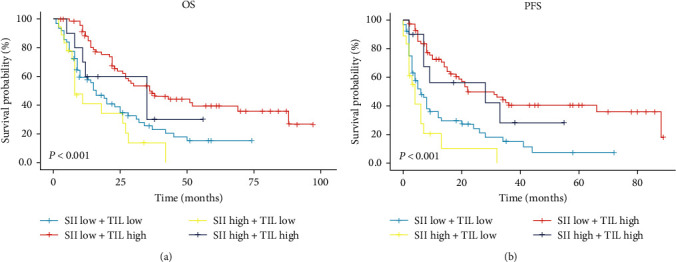
Kaplan–Meier survival curve of combination prognostic role of SII and TIL. (a) OS in different combinations was evaluated by TIL concentration and SII score. (b) PFS in different combinations was evaluated by TIL concentration and SII score. OS, overall survival; PFS, progression-free survival; TIL, tumor-infiltrating lymphocytes; SII, systemic immune-inflammation index.

It is well known that the occurrence and development of cancer are regulated by the interaction of several factors and processes. Previous results have demonstrated the important role of SII and TIL in clinical outcomes. However, it is not yet clear whether the combination of the two may show a high predictive value for evaluating clinical prognosis. We divided the two variables into different observation cohorts, including SII^low^ + TIL^low^, SII^low^ + TIL^high^, SII^high^ + TIL^low^, and SII^high^ + TIL^high^. The median survival time of the SII^low^ + STIL^high^ cohort group was the longest of all cohorts, reaching 36 months. The 1-, 3-, and 5-year OS rates were 88.0%, 50%, and 39.3%, respectively. As expected, the SII^high^ + TIL^low^ cohort had the shortest median survival of 8 months. The 1-, 2-, and 3-year OS rates were 41.0%, 34.2%, and 13.7%, respectively (Figure 6(a)). In addition, similar results appeared in the PFS assessment. The median PFS time in the SII^low^ + TIL^high^ cohort was 22 months, with 1-, 3-, and 5-year PFS rates of 72.3%, 50.0%, and 40.4%. The SII^high^ + TIL^low^ cohort was 4 months, 1-, 2-, and 3-year PFS rates of 20.6%, 10.3%, and 0%, respectively (Figure 6(b)).

## 4. Discussion

In this study, we investigated the correlation of clinical outcome with SII in patients who received CCRT. It was also a preliminary exploratory study to assess the association with SII based on different status of TIL. Studies have shown that SII calculated by collecting hematologic parameters before treatment was a reliable indicator of prognosis. Then, the TIL analysis also found a higher mortality rate in TIL patients. In addition, this study also clarified that SII was significantly associated with TIL. The combination of SII and TIL was a potential predictor of clinical outcomes in patients with EC. This indicates that the systemic inflammatory state and primary tumor TIL are interconnected and dynamically balanced.

As an important component and essential mediator of cancer carcinogenesis, systemic inflammatory conditions play a key position in the biological behavior of cancer [12, 23]. Besides, SII can be characterized and quantified by peripheral blood cell counts. It is precisely because of its convenient method of access that has received much attention in clinical practice, including EC. In the neoadjuvant phase, previous studies have shown that SII could act as an independent prognostic factor and found that SII > 644 was significantly associated with diminishing of OS [19]. Another study reported that high PLR and SII were significantly associated with pathological complete response [24, 25]. A significant association between low NLR and the occurrence of grade ≥ 3 hematologic toxicity was also observed [25]. In patients treated with surgical resection, a meta-analysis of 3,565 subjects showed that high SII was an independent predictor of poor OS, PFS, and cancer-specific survival in patients with ESCC [26]. In ESCC groups receiving radiotherapy (RT), the SII ratio of pre/post-RT, and mid-RT were potential markers for predicting clinical outcomes [27].

In our study cohort, we included patients with EC receiving CCRT. The median OS reached 25 months, and the 5-year survival rate was 28.0% in low SII. This was much longer than the 12 months for the high group, consistent with previous reports. There was a wide variation in determining the optimal threshold for SII according to the current study. Most results were reported to be between 583.45 and 792.49 [19, 24, 25, 28]. In our study, the optimal cut-off value for SII was defined as 1036.6, which was different from previous literature reports. The main reason is mainly related to the clinical stage of the patient. We note that previous valuable studies have focused on patients with surgically removed primary tumors. It significantly determines the size of tumor or the condition of lymph nodes at an earlier stage. This means that the SII values for this population are in the relatively low range. However, this study cohort was selected from patients with advanced stages, as this cohort accounts for the majority of EC in clinical practice. For reference, in this study, T4 accounted for 45.6%, N2/3 37.2%, and M1 19.4%. To some extent, increased tumor burden is associated with higher levels of systemic inflammation status.

TIL is the key executive unit of tumor–host immune interactions and represents the major breakthroughs in the study of immunogenic determinants and immunotherapeutic approaches [29, 30]. Due to the importance of the immune system in cancer surveillance and treatment, high TIL in the neoadjuvant treatment phase have been shown to predict response to chemotherapy and are also associated with survival benefit [31–33]. An increasing number of studies have also demonstrated the predictive role of TIL in clinical prognosis, and TIL concentrations were proportional to survival time [34–37]. In our study, we observed that the median time was more than two times OS (HR: 2.42) and five times PFS (HR: 3.05) in high TIL compared with low TIL. This further confirms the potential value of TIL in prognosis. Although 15% was used as the analysis threshold in this study, a similar previous study reported comparable results [38]. Related studies have reported that a threshold value of 50% is a better choice [39, 40]. However, 50% of TIL was not considered in this study, mainly because the concentration of TIL varies by cancer type. Only a few patients with EC had such a high percentage of TIL, more than a third of patients in our cohort had no detectable TIL in the primary tumor. In addition, it was observed that only 3.75% of patients achieved 50% of the TIL. Moreover, heterogeneity of research subjects between studies is also an inevitable factor in the existence of those differences.

Analyzing the formula for calculating the inflammatory index, lymphocytes were included as the main element in all indicators. Besides, lymphocytes in the tumor microenvironment are the main representatives of antitumor immunity. This led us to hypothesize that systemic inflammatory status and lymphocyte response may influence prognosis through local tumor immunity. Indeed, our finding of a strong relationship between SII and TIL also reinforced this hypothesis. To comprehensively show the distribution of the inflammatory index in TIL, TIL have been divided into absent (0%), mild (5%–15%), moderate (20%–60%), and strong (70%–80%) subgroups. Research have shown that the distribution of SII, PLR, and NLR were negatively associated with the TIL status, while LMR presented a positive correlation. This conclusion was also confirmed in breast cancer patients, where low NLR and high TIL were associated with a better prognosis and easier obtained to pathological complete response [41, 42]. In the prognostic analysis, groups were classified according to SII and TIL values, and clinical outcomes were evaluated. It was observed that SII^low^+ TIL^high^ had the best prognosis of all combinations, with a median OS and PFS of 36 and 22 months, respectively. The worst prognosis was identified as SII^high^+ TIL^low^, with a median OS and PFS of only 8 and 4 months. The other groups were somewhere in between. In addition, when the high SII and low TIL variables were evaluated separately, the 3-year OS rates were 17.8% and 22.9%, but together they decreased to 13.7%. A combined assessment of SII and TIL appears to be feasible in patients with EC. These data strongly support a relationship between systemic inflammatory status and local immunity. It is suggested that TIL function in a hypersensitive inflammatory environment may be impaired, further reflecting the poor clinical benefit.

## 5. Conclusion

This study confirmed the clinical prognostic value of SII in EC patients treated with CCRT and clarified that TIL status was negatively correlated with the distribution of SII. Additionally, the combined analysis was significantly better than either of the SII and TIL alone for evaluating clinical outcomes. Our study also has limitations: first, most of the patients included in the study belonged to the late stage, and whether it can be generalized to early-stage patients remain to be demonstrated. However, in patients with EC, early-stage patients represent only a small fraction of the real world, and the emphasis on advanced-stage patients is closer to clinical practice. Second, there are many TIL members, and H&E staining alone can only provide a rough estimate. More detailed immunohistochemical and molecular pathological tests are needed to investigate the underlying mechanism. While given its rapid and efficient properties are still widely used in clinical practice. In addition, given the relatively small number of cases in the high group of the SII prognosis model, there may cause bias. The study results are for reference only and are not recommended to other centers for use directly in clinical practice. Future studies could increase the sample size or conduct multicenter studies to confirm this conclusion.

## Figures and Tables

**Table 1:** Baseline clinicopathological characteristics according to SII.

Characteristic	Overall	SII		*P*-value
		Low (<1036.6)	High (≥1036.6)	
*n*	160	133	27	
Gender, *n* (%)				0.651
Female	38 (23.8%)	33 (20.6%)	5 (3.1%)	
Male	122 (76.2%)	100 (62.5%)	22 (13.8%)	
Age, *n* (%)				1.000
<70	111 (69.4%)	92 (57.5%)	19 (11.9%)	
≥70	49 (30.6%)	41 (25.6%)	8 (5%)	
ECOG, *n* (%)				1.000
1	18 (11.2%)	15 (9.4%)	3 (1.9%)	
2	142 (88.8%)	118 (73.8%)	24 (15%)	
Node size (cm), *n* (%)				0.192
<2.2	142 (88.8%)	120 (75%)	22 (13.8%)	
≥2.2	18 (11.2%)	13 (8.1%)	5 (3.1%)	
Length (cm), *n* (%)				0.016
<8.4	142 (88.8%)	122 (76.2%)	20 (12.5%)	
≥8.4	18 (11.2%)	11 (6.9%)	7 (4.4%)	
cT, *n* (%)				0.482
1	2 (1.2%)	1 (0.6%)	1 (0.6%)	
2	13 (8.1%)	12 (7.5%)	1 (0.6%)	
3	72 (45%)	59 (36.9%)	13 (8.1%)	
4	73 (45.6%)	61 (38.1%)	12 (7.5%)	
cN, *n* (%)				0.260
0	40 (25%)	37 (23.1%)	3 (1.9%)	
1	60 (37.5%)	48 (30%)	12 (7.5%)	
2	45 (28.1%)	35 (21.9%)	10 (6.2%)	
3	15 (9.4%)	13 (8.1%)	2 (1.2%)	
M, *n* (%)				0.081
No	129 (80.6%)	111 (69.4%)	18 (11.2%)	
Yes	31 (19.4%)	22 (13.8%)	9 (5.6%)	
Radiotherapy, *n* (%)				1.000
<50	15 (9.4%)	13 (8.1%)	2 (1.2%)	
≥50	145 (90.6%)	120 (75%)	25 (15.6%)	
Chemotherapy cycle, *n* (%)				1.000
<2	85 (53.1%)	71 (44.4%)	14 (8.8%)	
≥2	75 (46.9%)	62 (38.8%)	13 (8.1%)	
cCR, *n* (%)				0.205
No	111 (69.4%)	89 (55.6%)	22 (13.8%)	
Yes	49 (30.6%)	44 (27.5%)	5 (3.1%)	
TIL, *n* (%)				0.205
High (≥15%)	80 (50%)	70 (43.8%)	10 (6.2%)	
Low (<15%)	80 (50%)	63 (39.4%)	17 (10.6%)	
PLR, *n* (%)				0.005
<100	36 (22.5%)	36 (22.5%)	0 (0%)	
≥100	124 (77.5%)	97 (60.6%)	27 (16.9%)	
NLR, *n* (%)				< 0.001
<2.32	76 (47.5%)	76 (47.5%)	0 (0%)	
≥2.32	84 (52.5%)	57 (35.6%)	27 (16.9%)	
LMR, *n* (%)				<0.001
<2.5	22 (13.8%)	7 (4.4%)	15 (9.4%)	
≥2.5	138 (86.2%)	126 (78.8%)	12 (7.5%)	

SII, systemic immune-inflammatory index; TIL, tumor-infiltrating lymphocytes; PLR, platelet-to-lymphocyte ratio; NLR, neutrophil-to-lymphocyte ratio; LMR, lymphocyte-to-monocyte ratio; ECOG, Eastern Cooperative Oncology Group; cT, clinical tumor stage; cN, clinical nodes stage; M, metastasis; cCR, clinical complete response.

## Data Availability

The datasets used in the current study are available from the corresponding author upon reasonable request.

## Abbreviations

EC: Esophageal cancer
ESCC: Esophageal squamous cell carcinoma
CCRT: Concurrent chemoradiotherapy
SII: Systemic immune-inflammation index
PLR: Platelet-to-lymphocyte ratio
NLR: Neutrophil-to-lymphocyte ratio
LMR: Lymphocyte-to-monocyte ratio
TIL: Tumor-infiltrating lymphocytes
OS: Overall survival
PFS: Progression-free survival
HR: Hazard ratio
CI: Confidence intervals
ICIs: Immune checkpoint inhibitors
AJCC: American Joint Committee on Cancer
T: Tumor
N: Node
M: Metastasis
ECOG: Eastern Cooperative Oncology Group
cCR: Clinical complete response
RT: Radiotherapy.
